# Colossal positive magnetoresistance in surface-passivated oxygen-deficient strontium titanite

**DOI:** 10.1038/srep10255

**Published:** 2015-05-15

**Authors:** Adrian David, Yufeng Tian, Ping Yang, Xingyu Gao, Weinan Lin, Amish B. Shah, Jian-Min Zuo, Wilfrid Prellier, Tom Wu

**Affiliations:** 1 Division of Physics and Applied Physics, School of Physical and Mathematical Sciences, Nanyang Technological University, Singapore 637371, Singapore; 2 School of Physics, National Key Laboratory of Crystal Materials, Shandong University, Jinan, Shandong 250100, P. R. China; 3 Singapore Synchrotron Light Source, National University of Singapore, 5 Research Link, Singapore 117603, Singapore; 4 Shanghai Synchrotron Radiation Facility (SSRF), Shanghai Institute of Applied Physics, Chinese Academy of Sciences, Shanghai 201204, P. R. China; 5 Materials Science and Engineering and Materials Research Laboratory, University of Illinois, Urbana-Champaign 1304 W Green Street Urbana, IL 61801, USA; 6 Laboratoire CRISMAT, ENSICAEN, CNRS UMR 6508, 6 Boulevard Maréchal Juin, F-14050 Caen Cedex, France; 7 Physical Sciences and Engineering Division, King Abdullah University of Science and Technology, Thuwal 23955-6900, Saudi Arabia

## Abstract

Modulation of resistance by an external magnetic field, i.e. magnetoresistance effect, has been a long-lived theme of research due to both fundamental science and device applications. Here we report colossal positive magnetoresistance (CPMR) (>30,000% at a temperature of 2 K and a magnetic field of 9 T) discovered in degenerate semiconducting strontium titanite (SrTiO_3_) single crystals capped with ultrathin SrTiO_3_/LaAlO_3_ bilayers. The low-pressure high-temperature homoepitaxial growth of several unit cells of SrTiO_3_ introduces oxygen vacancies and high-mobility carriers in the bulk SrTiO_3_, and the three-unit-cell LaAlO_3_ capping layer passivates the surface and improves carrier mobility by suppressing surface-defect-related scattering. The coexistence of multiple types of carriers and inhomogeneous transport lead to the emergence of CPMR. This unit-cell-level surface engineering approach is promising to be generalized to others oxides, and to realize devices with high-mobility carriers and interesting magnetoelectronic properties.

Transition-metal oxides exhibit rich phase diagrams and exotic physical properties as a result of the complex interactions between multiple degrees of freedom^1^. As a prototypical transition metal oxide, SrTiO_3_ (STO) is a band insulator with a wide bandgap of 3.2 eV, while doping with oxygen vacancies or ions like La and Nb can induce an insulator-metal transition in STO at fairly low electron concentrations^2^. In addition, relaxor ferroelectricity was discovered in STO, which can be tuned by substrate strain^3^,^4^. Recently, the progress in growing oxide films with atomic scale controls has enabled the exploration of functional oxides beyond the conventional research on bulk samples. In particular, interesting transport phenomena were discovered at the polar-nonpolar LaAlO_3_/SrTiO_3_ (LAO/STO) interface^5^,^6^,^7^,^8^,^9^,^10^,^11^,^12^. Even room temperature deposition of gamma-alumina on STO was reported to lead to the formation of high-mobility electron gas^13^, indicting a strong reduction tendency. All these discoveries make STO the workhorse in the oxide electronics although challenges related to charge-trapping defects and low carrier mobility remain. In a general perspective, there is a strong need to take advantage of the strong structure-composition-property relationship in such transition-metal oxides to achieve high-performance devices with optimal properties.

In this work, we developed a surface-passivation approach to improve the carrier mobility in the prototypical oxide STO, and in particular we discovered colossal positive magnetoresistance (>30,000% at 2 K under a magnetic field of 9 T) in oxygen-deficient STO single crystals coated with STO/LAO bilayers. The colossal positive magnetoresistance (CPMR) observed here, to our knowledge, is the highest ever reported for oxide materials. Since the growth takes place purposely at low oxygen pressures, oxygen vacancies are generated in STO bulk as electron donors. Furthermore, the top LAO thin layer passivates the STO surface and contributes to the enhanced carrier mobility. Our analysis suggests that the observed CPMR is related to the high carrier mobility and multi-channel conduction in the surface-engineered oxygen-deficient STO, pointing out an effective surface engineering route towards high-mobility oxide magneto-electronics.

## Results

**Figure 1:**
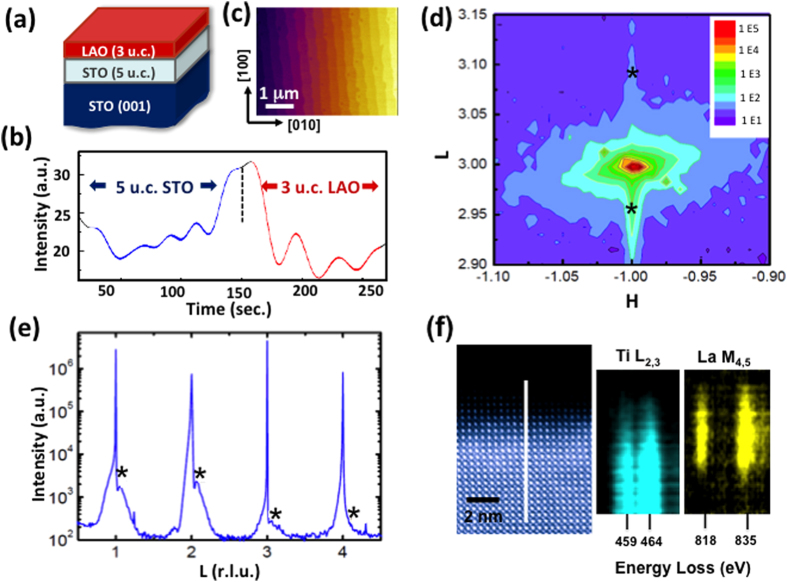
Synthesis and structural characterization of STO/LAO bilayers. (**a**) Schematic of the STO/LAO bi-layers grown on an oxygen-deficient STO single crystal. (**b**) RHEED oscillations recorded during the growth of the sample L-5/3 (5 u.c. STO and 3 u.c. LAO layers sequentially grown on a TiO_2_-terminated STO substrate at an oxygen pressure of 10^-6^ mbar and 800 °C). (**c**) AFM image of L-5/3 showing the step-terrace structure. (**d**) Asymmetric 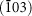 RSM data. The two stars mark the positions of LAO (upper) and STO (lower) diffraction peaks. Both layers are fully strained in the basal plane of STO substrate. (**e**) Crystal truncated rod (*l* scan) data of the sample L-5/3. The stars on the right of the sharp substrate peaks mark the LAO peaks, while the shoulders on the left originate from the STO homoepitaxial layer. (**f**) Cross-sectional STEM image (left) and EELS line scans (right) of the sample L-5/3.

The schematic of the surface-engineered STO single crystals is shown in Fig. 1(a). It is well known that the low-pressure PLD growth generates high-density oxygen vacancies in the surface layer of the STO substrates. For growing reference samples, we used a higher oxygen pressure of 10^-3^ mbar. The film thickness was monitored *in situ* by RHEED, and the intensity oscillation confirms a layer-by-layer growth mode (Fig. 1(b)) The LAO layer is fixed at 3 u.c., and the thickness of the homoepitaxial STO layers is varied. We purposely set the thickness of the LAO layer below the critical value for the onset of two-dimensional electron gas (2DEG) at the LAO/STO interface, thus the conduction in our sample mainly originates from the electrons in the STO bulk donated by oxygen vacancies generated during the low-pressure PLD growth^8^. The samples are denoted as L-n/3 (“L” stands for the low oxygen pressure, while n and 3 are the numbers of u.c. in the STO homoepitaxial layer and the LAO capping layer, respectively). The atomic force microscopy (AFM) image in Fig. 1(c) taken on the sample L-5/3 suggests that the surface is featured by u.c.-high steps. In this particular sample, the miscut orientation of the steps is ~6° away from the [010] direction of the STO single crystal substrate and the width of terraces is ~0.5 μm.

In sample L-5/3, a typical bi-layer, X-ray reciprocal space mapping (RSM) data (Fig. 1(d)) suggest a coherent growth, i.e. both the STO and LAO layers have the same in-plane lattice parameter as the STO substrate. Figure 1(e) shows the scan data along **(00l)** crystal truncation rod; we estimated a small lattice contraction in LAO with c = 3.77 Å and an out-of-plane lattice expansion in the STO layer with c = 3.93 Å. The slight modification of the LAO lattice parameter may be attributed to elastic deformation and electrostrictive effect^14^. The larger out-of-plane STO lattice parameter of L-5/3 compared to the bulk value (3.905 Å) was previously reported^15^,^16^, which may have multiple origins like oxygen vacancies, change of Ti valence, and cation off-stoichiometry. Particularly, it was reported that 1% of Sr deficiency in STO causes 0.06 Å expansion of the c axis^17^.

The sample was further examined by aberration-corrected STEM (JEOL 2200FS) in the cross-sectional viewing geometry (Fig. 1(f)). The atomic resolution Z-contrast image of Sample L-5/3 obtained using a high angle annular dark field detector (HAADF) shows an epitaxial heterostructure. The cation intermixing near the interface region was investigated using EELS. Figure 1(f) shows the EELS line scans for the La M4,5 and Ti L2,3 edges across the bilayer and the substrate. La was observed to diffuse into the STO film and the STO substrate, extending over 6 nm, which is much more than the nominal thickness of the LAO layer (3 u.c. or 1.1 nm). On the other hand, Ti is absent in the topmost layer of 1-2 nm (i.e., the LAO overlayer), suggesting that the B-site Al-Ti exchanges is less significant than the A-site La-Sr exchange in the heterostructure. It has been reported that compared to the abrupt interface configuration, cation exchanges at the LAO/STO interface reduce the overall dipole-related energy. Furthermore, the doping of La in the STO probably reduces the effective mass of electrons and further enhances the conduction^20^.

These results of structural characterization on surface-engineered STO single crystal suggest very high crystalline qualities of the samples. The surfaces of as-received STO substrates are often featured with various kinds of defects associated with mechanical cutting, polishing and chemical etching. These structural imperfections on the surfaces reduce the mobility of carriers and compromise the performance of oxide electronic devices. The goal of growing unit-cell-controlled bilayers on STO single crystals with tailored conditions is to retain and even improve the transport properties by reducing the surface-related scatterings and introducing carriers in high quality single crystals.

**Figure 2:**
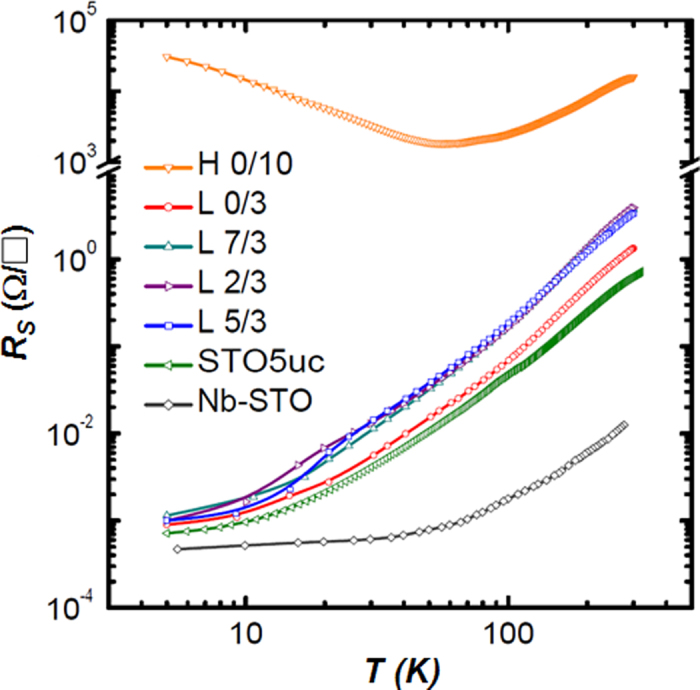
Transport properties of the STO/LAO bilayers. Temperature dependence of the sheet resistance suggests a metallic behavior in the n/3 samples. In contrast, the sheet resistance 10 u.c. LAO single layer grown on STO substrate (sample H-0/10) is much higher and shows an upturn feature at □60 K. For comparison, we also measured STO film (5 u.c.) grown on STO substrate (STO5uc) and Nb-doped STO single crystal substrate (Nb-STO).

The temperature-dependent sheet resistance (R-T) data on our low pressure grown samples present a metallic behavior (Fig. 2). Below 10 K, the R-T curves level off, and the sheet resistance at 5 K is 1 × 10^-3^ /□.The residual resistance ratio (RRR) = R(300 K)/R(5 K) measured on the sample L-5/3 reaches 3700, which is much larger than the values obtained in other doped STO single crystals^21^ and indicates a high sample quality. As shown in Fig. 2, we measured a Nb-doped (0.7 wt.%) STO substrate with dimensions of 5 × 5 × 0.5 mm^3^, and it shows a low sheet resistance of 1.6 × 10^-2^ W/□ at room temperature, but its RRR is only 300. This indicates that the carriers in the bilayer-covered samples experience less scattering by defects and impurities compared to the Nb-doped single crystal. The single-layer 3 u.c. LAO sample (L-0/3) and the STO/LAO bilayers with other STO thicknesses (L-0/3, L-2/3 and L-7/3) show a similar metallic transport behavior. On the other hand, the STO/LAO bi-layer samples deposited at a high oxygen pressure of 10^-3^ mbar, namely H-0/3, H-2/3 and H-5/3, remain highly insulating, which indicates that the electrons donated by the oxygen vacancies are responsible for the observed conduction in the low pressure grown samples. We also measured a 10 u.c. LAO layer deposited directly on STO substrates at a high pressure of 10^-3^ mbar (sample H-0/10), which shows a room temperature sheet resistance of 3.1 × 10^4^ /□; the upturn of resistance at 60 K is consistent with the previous reports^5^,^22^.

**Figure 3:**
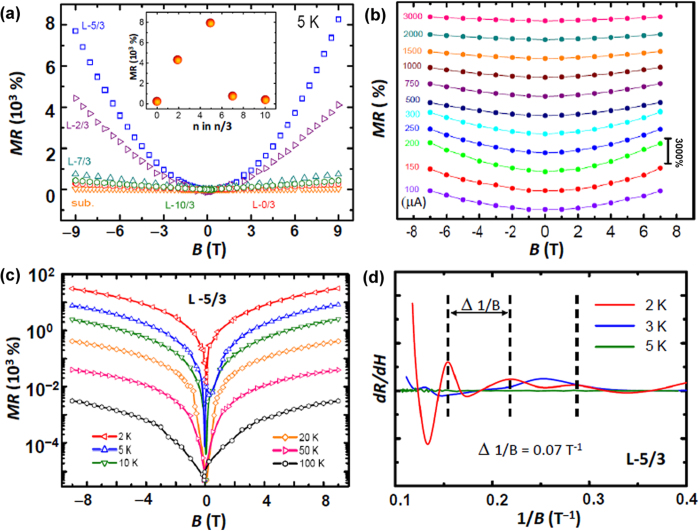
Magneto-transport data of the LAO/STO bi-layers. (**a**) MR data of the L-n/3 series of samples measured under a varying perpendicular magnetic field at 5 K. Inset compares the MR values measured at 5 K and 9 T for samples with various thickness of the LAO layer. The sample L-5/3 presents the largest MR of □8,000%. For comparison, a vacuum-annealed single crystal STO substrate was also measured. (**b**) Current-dependent MR measured at 5 K in the 5/3 sample. The largest effect occurs at a current of 200 μA. (**c**) Semi-logarithmic plot of the temperature-dependent MR measured on the sample L-5/3. At 2 K and 9 T, its MR reaches about 30,000%. (**d**) Numerical derivative of the resistance with respect to magnetic field is plotted versus the inverse of the field. Shubnikov-de Haas oscillations were observed at 2 K and 3 K. The vertical dashed lines mark the oscillation period of the 2 K data.

We investigated the magnetotransport properties of the samples, and MR is defined as (*R*(*H*)-*R*(*H*=0))/*R*(*H=*0)=∆*R*/*R.* As shown in Fig. 3(a), the LAO/STO bi-layer (samples L-5/3) exhibits CPMR, which reaches 8,000% at 5 K and 9 T. During the measurements, the magnetic field is applied perpendicular to the substrate plane, and we note that the in-plane MR is much smaller. For comparison, we annealed a reference STO single crystal substrate at 900 ^o^C under high vacuum (10^-6^ mbar) for 20 hours, and a MR of 40% was observed under the same measurement conditions. In previously reported Ar-ion-irradiated STO samples^23^,^24^, the low-temperature magnetoresistance falls in the range of 20 – 300%. Similarly, in the high-pressure-grown LAO/STO samples, the magnetoresistance is typically smaller than 100%^25^,^26^,^27^. We also found that the magnitude of CPMR in the samples sensitively depends on the thickness of the homoepitaxial STO layer: it is smaller in L-2/3 (4,400%), and drastically decreases in L-7/3 (750%), L-10/3 (450%) and L-0/3 (300%). The inset of Fig. 3(a) summarizes the dependence of magnetoresistance on the thickness of the STO layer. In general, the growth of the STO layer improves the quality of the STO substrate surface. Furthermore, the thickness of the STO layer grown under a low oxygen pressure may be correlated with the distribution and characteristics of oxygen vacancies and associated carriers. On another aspect, the top LAO layer also plays an important role, and it is indispensable to achieve the highest MR. For comparison, we also measured the MR of a sample of 5 u.c. STO layer grown on STO (001) substrate, and the value is around 450%. This top LAO layer effectively separates the carriers from the scattering centers on the sample surface such as polar molecules or other absorbates. In this way, the LAO layer plays the role of a capping layer to preserve high-mobility carriers.

In addition, the observed CPMR exhibits a strong dependence on the measurement current (Fig. 3(b)), and the highest MR in the sample L-5/3 occurs at 200 □μA. The nonmonotonous current dependence of MR excludes the heating effect as the origin since a larger current is always expected to cause more heating. Recently, very large positive magnetoresistance was also observed in silicon devices, which was attributed to the neutrality breaking of the space-charge effect as a result of charge injection^28^. In the silicon devices, a similar nonmonotonous current dependence was observed in the MR measurements, and the complementary modulation by electric field and magnetic field potentially provides new mode of device operation.

The so-called inhomogeneous magnetoresistance (IMR) was also observed in other non-magnetic materials like silver chalcogenide and indium antimonide, which was ascribed to the inhomogeneous doping and the large spatial fluctuation in conductivity^29^,^30^. The similar current dependence observed in our samples suggests that inhomogeneous distribution of carriers may play an important role in the observed novel magnetotransport properties. This conduction inhomogeneity is different from the recently proposed electronic phase separation scenario^27^. On the other hand, compositional variation^31^ and inhomogeneous distribution of electron-donating oxygen vacancies may play important roles.

We also measured the magnetoresistance of the sample L-5/3 as a function of temperature. As shown in Fig. 3(c), with a measurement current of 200 μA, its MR is as high as 30,000% at 2 K and 9 T. To our knowledge, it is the highest positive MR ever reported on oxide materials. The magnetoresistance decreases rapidly as the measurement temperature goes up; it is 400% at 20 K and 3% at 100 K. This hints a correlation between the CPMR and the high carrier mobility often observed at low temperatures in such samples.

Notably, the sample L-5/3 showed the Shubnikov de Haas (SdH) oscillations, indicative of high-mobility carriers. Figure 3(d) plots the numerical derivative of the resistance with respect to the inversed magnetic field. At 2 K, the pronounced oscillation appears periodic as a function of 1/B. The oscillation at 3 K becomes much weaker with anomalous features, and it disappears at 5 K. We should note that only electrons with the highest mobility in the highest lying subband contribute to the SdH oscillation, and the density of these electrons is usually much smaller than the total electron density^32^. The period of the SdH oscillation, Δ (1/B), can be used to directly estimate the area of the Fermi surface (A) that is defined as the surface of constant energy of the highest occupied state in k-space^33^,^34^. In the case of the L-5/3 sample, the oscillation period is 14.3 T at 2 K, thus A is estimated as 1.36 × 10^13^ cm^-2^, corresponding to a Fermi wave vector k_F_ of 2.08 × 10^6^ cm^–1^ if a spherical Fermi surface and a parabolic band are assumed. Finally, we obtained the electron density n = k_F_^3^/ 3π^2^ = 3.04 × 10^17^ cm^–3^, which is comparable to the value obtained previously in the LAO/STO samples grown at low pressures^33^.

## Discussion

**Figure 4:**
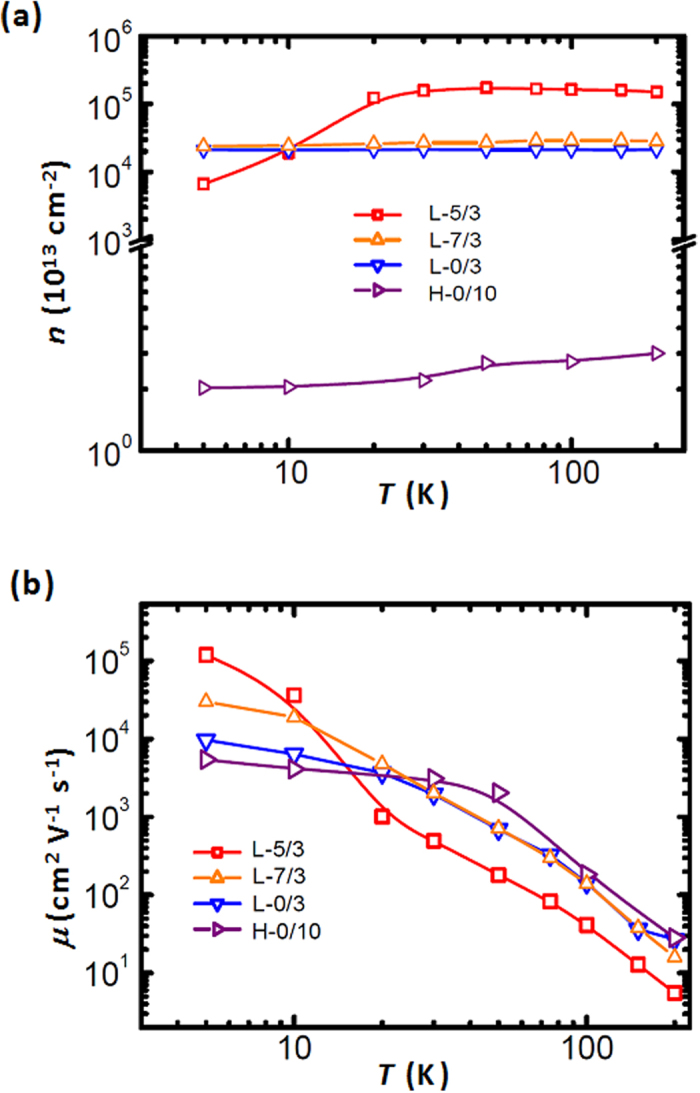
Results of Hall effect measurements. (**a**) Temperature-dependent carrier density data of the samples L-0/3, L-5/3 and L-7/3 calculated form the Hall Effect measurements. For comparison, the carrier density data of the sample H-0/10 are also shown. (**b**) Corresponding mobility data of the samples.

The observed CPMR effect and the current dependence favor the IMR model developed by Parish and Littlewood for inhomogeneous conducting media, where resistor networks were used to mimic the transport in such systems^35^,^36^. According to the model, the MR is dominated either by the average mobility, <μ>, or by the width of the mobility distribution, Δμ. In particular, for a narrow mobility distribution, MR is expected to be a function of the effective carrier mobility^35^. To verify the mobility-related scenario, we used Hall effect measurements to extract the characteristics of the carriers. As shown in Fig. 4(a), the sheet carrier densities of the samples L-0/3 and L-5/3 were recorded as 2.1 × 10^17^ cm^-2^ and 6.6 × 10^16^ cm^-2^, respectively, which are the same order of magnitude as that of doped STO single crystals^21^. In contrast, the carrier density of the high-pressure grown sample H-0/10 is three to four orders of magnitude lower. As shown in Fig. 4(b), the sample L-5/3 presents the highest value of mobility (1.18 × 10^5^ cm^2^ V^-1^ s^-1^) measured at 5 K. The samples L-0/3 and L-7/3 exhibit lower mobility values of 9.6 × 10^3^ and 3.04 × 10^4^ cm^2^V^-1^s^-1^, respectively. In comparison, the high-pressure sample H-0/10 exhibits the lowest mobility. This is expected because electrons in the LAO/STO interface samples grown at high oxygen pressure typically exhibit low mobility^6^,^9^,^25^,^37^,^38^, i.e., between 3.2 × 10^2^ and 2.4 × 10^3^ cm^2^ V^-1^ s^-1^. It is noteworthy that the mobility of L-5/3 is even higher than the results of STO single crystals doped by either oxygen vacancies or Nb ions. One of the reasons may be related to smoother interfaces with smaller roughness in such surface engineered oxide heterostructures. Previous scanning tunneling microscopy studies have shown that homoepitaxial STO thin films can significantly improve the surface roughness of STO substrates and induce regular (2 × 2) surface reconstruction^39^, which may reduce the surface- and interface-related scattering and contribute to the observed high-mobility transport. The high mobility of the carriers in turn contributes to the CPMR effect observed in the surface modified STO samples.

It is clear that the conduction mechanism in our samples is different from the two-dimensional electron gas produced in the conventional “high”-pressure-grown LAO/STO heterostructures. In particular, the conduction layer in our “low”-pressure-grown surface-modified STO single crystal samples is much thicker than the 2DEG at the LAO/STO interface, and thus the transport in such surface-modified samples should have a three-dimensional nature. Furthermore, the transport properties of the samples were reliably controlled by the synthesis parameters, particularly the growth temperature, the oxygen pressure, and the growth time. We found that two different batches of sample, prepared separately in laboratories in Singapore and France, presented very similar magneto-transport properties. Importantly, our synthesis approach is effective at mitigating the undesired carrier scattering in the surface region and producing high-mobility electrons in an oxide single crystal.

In addition, there are multiple conducting channels in the transport of our samples. One channel with very high carrier mobility but a small density contributes to the observed SdH oscillation. The majority of the carriers are distributed in a rather thick slab underneath the LAO top layer. The measured sheet carrier density from the Hall effect is on the scale of 10^16^ - 10^17^ cm^-2^, consistent with the previous reports^40^. If we assume a bulk carrier density of 10^18^ cm^-3^, then the carriers are distributed in a thickness of 0.01 – 0.1 cm. If we use the bulk carrier density of 3 × 10^17^ cm^-3^ obtained from the SdH oscillation, then we arrive at a thickness of 0.03 – 0.3 cm for the conduction layer. In other words, the transport in our low-temperature-grown samples has a clear three-dimensional bulk-like character. However, we cannot exclude the existence of 2DEG at the interface since the formation of 2DEG was reported for not only LAO/STO interfaces^41^, but also vacancy-containing samples grown at low oxygen pressures^42^,^43^. Nevertheless, the total number of electrons in 2DEG should be much smaller than that of electrons in the bulk single crystal, and thus the transport is dominantly three-dimensional.

It is well recognized that growing a thin layer of LAO or other oxides at low oxygen pressures is more effective to generate carriers in STO compared to the vacuum annealing technique, and oxygen readily diffuses out of the bulk, leaving behind carrier-donating oxygen vacancies^33^. A recent *in situ* transport measurement demonstrated that the low-pressure PLD growth could facilitate efficient reduction of single-crystal STO^44^. Furthermore, the homoepitaxial nature of the growth STO layers prevents strain and reduces defect formation. However, as shown in Fig. 3(a), there is an optimal thickness of the STO layer in order to achieve good conduction; if the STO layer is too thick, the excessive scatterings of carriers by the vacancies may bring down the mobility, leading to the observed non-monotonous trend of MR as a function of the STO layer thickness.

Surface passivation is a well-know technique to produce high quality semiconductor devices, and it has been used, for example, to improve the carrier lifetime and the performance of crystalline Si solar cells^45^. In oxide heterostructures, both structural defects like oxygen vacancies and surface absorbates can degrade the transport and diminish the carrier mobility. Recently, an ultrathin strontium copper oxide layer was demonstrated to improve the properties of LAO/STO heterostructures, and the origin was attributed to the reduction of kinetic barrier for oxygen exchange and the reduced defect scattering^46^. In our work, STO single crystals with high crystalline quality serve as the host, and the deposition of STO/LAO bilayers introduces the high-mobility carriers in a controlled manner. Furthermore, the capping LAO layer may help separate the carriers from the surface scattering centers, providing passivation for the samples. This approach is similar to the recent report of record high mobility (1.4 × 10^5^ cm^2^ V^-1^ s^-1^ at 2 K) observed at the interface between epitaxial alumina films and STO single crystals^47^. It will be interesting to check if this surface passivation strategy works for other conducting oxide single crystals, thin films, and heterostructures.

**Figure 5:**
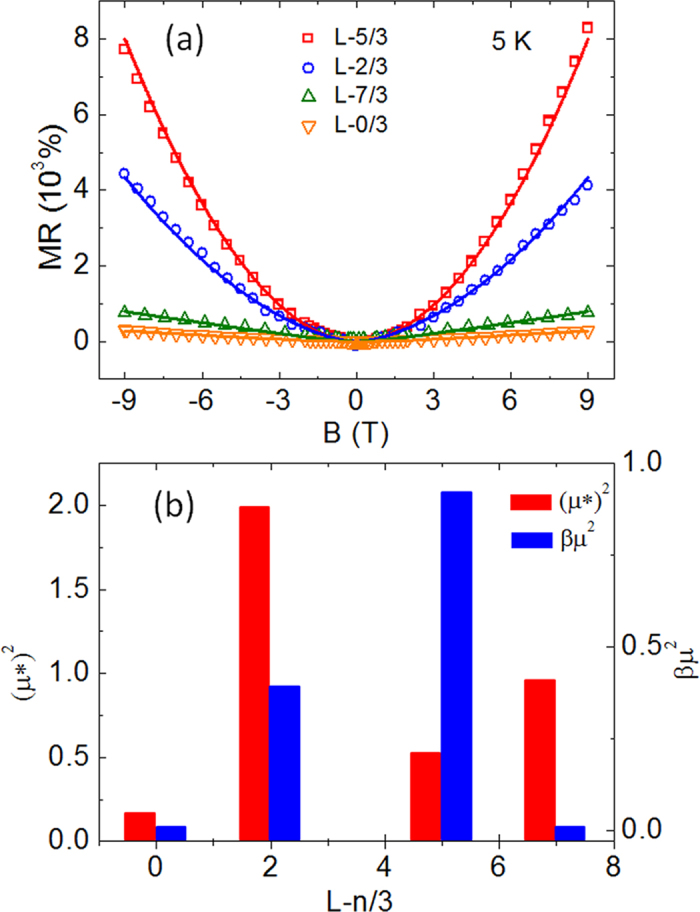
Fitting to the CPMR. (**a**) MR data (open symbols) of the L-n/3 series of samples measured at 5 K along with the theoretical fittings (solid lines) according to the phenomenological model. (**b**) Corresponding fitting parameters with a unit of 1/T^2^ derived for different samples.

The characteristics of the observed CPMR including the linear behavior at high magnetic field without saturation and the nonmonotonous dependence on the measurement current indicate the crucial role of inhomogeneity in the magnetotransport. The IMR can be described by the resistor network model that was developed to mimic the transport in such highly inhomogeneous systems^35^. On the other hand, the geometrical magnetoresistance due to the Lorentz force gives rise to the quadratic field dependence of MR at low magnetic field^48^. Herein, we proposed an empirical model to describe the magneto-transport characteristics, particularly the positive nonsaturating magnetoresistance observed in the surface-engineered STO single crystals. In this phenomenological model, we combine the classical magnetotransport model of inhomogeneous conductors with the Lorentz magnetoresistance effect. The magnetic field dependence of the sample resistance is given by


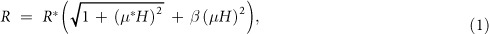


where R^*^ is sample dependent effective resistance, μ^*^ and *μ* are, respectively, the effective mobility and the carrier mobility, and *β* is a dimensionless coefficient. In the contribution derived from the random resistor network, R^*^ and μ^*^ are different from those of the resistor elements that generate the network; instead they depend on the geometry of the resistor elements as well as the connections between elements^35^. Good agreement is obtained between the theoretical fittings and the experimental data, as shown in Fig. 5(a), suggesting that both the inhomogeneity-related magnetoresistance and the Lorentz-type magnetoresistance play crucial roles. The corresponding fittings parameters are given in Fig. 5(b). Although the sample L-5/3 does not exhibit the highest effective mobility μ^*^, its electron mobility *μ* is much higher than those of other samples. This result indicates that both carrier mobility and electronic inhomogeneity are critical parameters for producing the CPMR behavior.

As an important feature, the operation of our devices does not rely on the presence of any magnetic material, and unlike the conventional MR devices which relay on the switching of individual low-coercivity magnetic layers^49^,^50^, our devices are suitable for sensing operations at very high magnetic fields on the order of Tesla. We should also note that the colossal magnetoresistance effect in mixed-valance manganites is negative in sign, and the mechanism hinges on magnetic field tuning of metal-insulator transition^51^, which is different from our case. The results reported herein open doors for exploring CPMR in high-mobility non-magnetic transition metal oxides with multi-channel electronic transport. As shown in Fig. 4(a), since there is no carrier “freeze out” at low temperatures, these oxide devices may be suitable for the space applications at cryogenic temperatures.

In summary, we introduced in this work a novel surface-passivation approach to produce extraordinary magneto-transport properties in degenerate semiconducting bulk STO, a prototypical electronic oxide. Tailoring the thickness of the STO homoepitaxial layer and the LAO layer with the u.c. precision in a highly reducing growth environment is the key to achieving the high carrier mobility and CPMR in the STO single crystals. This surface-passivation approach effectively improves the overall mobility of transport carriers in semiconducting bulk STO. Such a strategy of simultaneous carrier doping and surface engineering may be applicable to other functional oxides with strong structure-property correlations, facilitating the developments of high-performance oxide devices for electronic, thermoelectric and energy harvesting applications.

## Methods

STO/LAO bilayers were grown on TiO_2_-terminated (001) STO substrates (Shinkosha co., Japan) using pulsed laser deposition (PLD) at a temperature of 800 ^o^C and an oxygen pressure of either 10^-6^ mbar (low pressure samples) or 10^-3^ mbar (high pressure samples). The synthesis conditions are similar to our previous reports^52^,^53^,^54^,^55^. The substrates have typical dimensions of 5 × 5 × 0.5 mm^3^. The frequency of the excimer KrF laser (248 nm) was 1 Hz, and the fluency was ~ 1 J/cm^2^. The number of unit cells of both the STO homoepitaxial layer and the LAO overlayer was controlled by reflection high-energy electron diffraction (RHEED). After growth, the samples were cooled in the growth pressure to room temperature with a rate of 5 ^o^C/min. In the sample notation, the first letter represents the deposition pressure (L and H stand for low and high oxygen pressures, respectively), which is followed by the numbers of unit cell (u.c.) in the STO homoepitaxial layer and the LAO capping layer.

High-resolution X-ray diffractometry (HR-XRD) were performed in the beamlines at Singapore Synchrotron Light Source (SSLS) and Shanghai Synchrotron Radiation Facility (SSRF). Scanning transmission electron microscopy (STEM) and electron energy loss spectroscopy (EELS) were performed at 200 kV using a JEOL2200FS electron microscope equipped with a CEOS probe corrector and an in-column energy filter. Samples for electron microscopy were prepared by wedge polishing and low energy ion milling. The EELS linescans were smoothed once in spatial dimensions for clarity. Conductivity of the samples was measured in a Physical Property Measurement System (Quantum Design) with four-point configuration. Indium wires were bonded directly to the electrodes. The direction of the current iwa kept along the [100] direction regardless of the miscut angles of the substrates. Unless specified otherwise, the measurement current was 200□μA. In general, the magnetic field was applied perpendicular to the substrate plane in the MR measurements. In the Hall effect measurements, the resistivity data was anti-symmetrized in order to exclude the longitudinal component.

## Additional Information

**How to cite this article**: David, A. *et al.* Colossal positive magnetoresistance in surface-passivated oxygen-deficient strontium titanite. *Sci. Rep.* **5**, 10255; doi: 10.1038/srep10255 (2015).
